# *Bradyrhizobium* *ottawaense* sp. nov., a symbiotic nitrogen fixing bacterium from root nodules of soybeans in Canada

**DOI:** 10.1099/ijs.0.065540-0

**Published:** 2014-09

**Authors:** Xiumei Yu, Sylvie Cloutier, James T. Tambong, Eden S. P. Bromfield

**Affiliations:** Agriculture and Agri-Food Canada, 960 Carling Ave, Ottawa, Ontario K1A 0C6, Canada

## Abstract

Sixteen strains of symbiotic bacteria from root nodules of *Glycine max* grown in Ottawa, Canada, were previously characterized and placed in a novel group within the genus *Bradyrhizobium*. To verify their taxonomic status, these strains were further characterized using a polyphasic approach. All strains possessed identical 16S rRNA gene sequences that were 99.79 % similar to the closest relative, *Bradyrhizobium liaoningense* LMG 18230^T^. Phylogenetic analysis of concatenated *atpD, glnII, recA, gyrB, rpoB* and *dnaK* genes divided the 16 strains into three multilocus sequence types that were placed in a highly supported lineage distinct from named species of the genus *Bradyrhizobium* consistent with results of DNA–DNA hybridization. Based on analysis of symbiosis gene sequences (*nodC* and *nifH*), all novel strains were placed in a phylogenetic group with five species of the genus *Bradyrhizobium* that nodulate soybeans. The combination of phenotypic characteristics from several tests including carbon and nitrogen source utilization and antibiotic resistance could be used to differentiate representative strains from recognized species of the genus *Bradyrhizobium*. Novel strain OO99^T^ elicits effective nodules on *Glycine max*, *Glycine soja* and *Macroptilium atropurpureum*, partially effective nodules on *Desmodium canadense* and *Vigna unguiculata*, and ineffective nodules on *Amphicarpaea bracteata* and *Phaseolus vulgaris*. Based on the data presented, we conclude that our strains represent a novel species for which the name Bradyrhizobium *ottawaense* sp. nov. is proposed, with OO99^T^ ( = LMG 26739^T^ = HAMBI 3284^T^) as the type strain. The DNA G+C content is 62.6 mol%.

Soybean [*Glycine max* (L.) Merrill] is an economically important grain legume that can form a nitrogen-fixing association with species of soil bacteria belonging to the genus *Bradyrhizobium*. In a previous study (Tang *et al.*, 2012), populations of symbiotic bacteria associated with soybeans at two field sites in eastern Canada were characterized on the basis of multilocus sequence analysis (MLSA) of six protein encoding (housekeeping) genes. Phylogenetic analyses resulted in the identification of several novel lineages within the genus *Bradyrhizobium*. One of these novel lineages was encountered at only one of the two field sites and consisted of a group of 16 strains. In this work, we further characterize these strains using a variety of genotypic and phenotypic methods, and, based on the results, a novel species, Bradyrhizobium *ottawaense* sp. nov. is proposed.

The 16 novel strains of symbiotic bacteria (Table S1, available in the online Supplementary Material) were obtained from root nodules of soybeans grown at a field site in Ottawa, Ontario (Tang *et al.*, 2012). Bacteria were grown on yeast-extract mannitol (YEM) agar medium (Vincent, 1970) and pure cultures maintained in 20 % (w/v) glycerol at −80 °C. Bacterial cells were Gram-stain-negative (Powers, 1995), and, based on the Schaeffer–Fulton staining method (Hussey & Zayaitz, 2012), were non-spore-forming. Colonies on YEM agar were mucoid, beige, translucent, circular and measured <1 mm in diameter after 7 days at 28 °C. Cell morphology was investigated using a transmission electron microscope (H-7000; Hitachi). Bacteria were cultured in stationary YEM broth for 2 days at 28 °C and stained with 1 % phosphotungstic acid (pH 7.0) (Hayat & Miller, 1990). All tested strains (OO99^T^, OM9 and OO85) had rod-shaped cells, subpolar flagella and a cell size (Fig. S1) that is consistent with the characteristics of the genus *Bradyrhizobium* (Garrity *et al.*, 2005). Cell motility was demonstrated using the semisolid medium puncture method (Shields & Cathcart, 2012) with bacteria grown on semisolid YEM agar medium. Production of an alkaline reaction on YEM agar containing bromothymol blue after 21 days at 28 °C (Bromfield *et al.*, 2010) and mean generation times (12–13 h) of bacterial cultures in YEM broth (Itakura *et al.*, 2008; Wittwer, 2014) (Table 1) were also typical of the genus *Bradyrhizobium* (Garrity *et al.*, 2005; Vincent, 1970).

**Table 1.** Phenotypic characteristics of Bradyrhizobium *ottawaense* sp. nov. strains OO99^T^, OM9 and OO85 and type strains of species of the genus *Bradyrhizobium* Strains: 1, *Bradyrhizobium ottawaense* sp. nov. OO99^T^; 2, *B. ottawaense* sp. nov. OM9; 3, *B. ottawaense* sp. nov. OO85; 4, *B. cytisi* LMG 25866^T^; 5, *B. yuanmingense* LMG 21827^T^; 6, *B. iriomotense* LMG 24129^T^; 7, *B. liaoningense* LMG 18230^T^; 8, *B. betae* LMG 21987^T^; 9, *B. japonicum* USDA 6^T^; 10, *B. elkanii* USDA 76^T^.

Characteristic	1	2	3	4	5	6	7	8	9	10
Carbon source utilization*										
d-Mannose	+	+	+	+	+	+	+	+	+	+
d-Xylose	±	+	±	+	+	+	+	+	+	+
Lactose	−	−	−	−	±	−	−	−	±	±
l-Arabinose	+	+	+	+	+	+	+	+	+	+
d-Glucose	±	±	+	+	±	+	−	+	+	+
d-Mannitol	+	−	+	+	+	−	−	+	+	+
Trehalose	−	−	−	−	−	−	−	−	−	−
Succinic acid	−	−	−	−	−	−	−	−	±	±
l-Sorbose	−	−	−	−	−	−	−	+	±	±
Maltose	±	±	±	−	±	−	−	+	+	+
l-Rhamnose	±	±	±	−	±	−	+	−	+	+
Nitrogen source utilization*										
l-Proline	±	±	±	±	+	−	−	+	+	+
l-Glutamic acid	±	±	±	±	+	±	+	+	+	+
Aspartic acid	−	−	−	±	−	±	±	−	−	−
Glycine	−	−	−	−	−	−	−	−	−	−
dl-α-Alanine	−	−	−	−	±	−	−	−	−	−
l-Threonine	+	+	+	+	+	±	−	+	+	+
Antibiotic resistance (µg ml^−1^)†										
Kanamycin	100	100	100	nt	25	<25	<25	<25	<25	nt
Tetracycline	20	20	10	nt	20	<5	10	10	20	nt
Chloramphenicol	100	100	100	nt	100	<5	<25	100	100	nt
Erythromycin	100	<50	50	nt	<50	200	<50	50	100	nt
Cefuroxime	<5	<5	<5	nt	<5	<5	<5	<5	30	nt
Penicillin	<10	<10	<10	nt	<10	<10	<10	50	50	nt
Growth at:										
1 % (w/v) NaCl	−	−	−	nt	−	−	−	−	−	nt
10 °C	−	−	−	nt	−	−	−	−	−	nt
37 °C	−	−	−	nt	+	−	−	−	−	nt
pH 5	+	+	+	nt	+	+	+	+	+	nt
pH 10	±	±	±	nt	+	−	−	+	+	nt
Acid/alkali production (pH)†,‡	7.36±0.04	7.45±0.13	7.34±0.06	nt	7.00±0.06	nt	nt	nt	nt	nt
Mean generation time (h)†	12.3±0.6	12.9±0.9	13.1±0.9	nt	14.1±1.6	nt	nt	nt	nt	nt

+, Positive; ±, weak; −, negative; nt, not tested.

* Values are based on duplicates.

† Values are based on five replicates.

‡ Uninoculated control, pH 6.76±0.06; *Ensifer meliloti* ATCC 9930^T^, pH 5.75±0.05.

Almost full-length 16S rRNA gene sequences were generated using primers 16Sa and 16Sb (van Berkum & Fuhrmann, 2000) and sequence alignment was carried out using the Infernal secondary-structure-based aligner implemented in the Ribosomal Database Project program version 11.1 (Cole *et al.*, 2014). Partial sequences of housekeeping (*atpD*, *glnII*, *recA*, *gyrB*, *rpoB* and *dnaK*) and symbiotic (*nodC* and *nifH*) genes were generated for the 16 novel strains of the genus *Bradyrhizobium* as well as for reference taxa not available in public databases. Preparation of genomic DNA, amplification, primers, nucleotide sequencing, sequence alignment and editing was as described previously (Tang *et al.*, 2012). The *dnaK* sequence of ‘B*radyrhizobium* retamae’ Ro19 required designing a second forward sequencing primer (FdnaK_SC1: GAGCAGCAGATCCGGATTCA) in order to obtain the 3′ end of the sequence. GenBank accession numbers of nucleotide sequences are given in Tables S1 and S2.

Bayesian phylogenetic analyses were carried out using MrBayes version 3.2.1 with default priors (Altekar *et al.* 2004). For each dataset, two concurrent analyses with four chains (three heated and one cold) were run for ten million generations with sampling every 2000 generations. Convergence was judged satisfactory when the average sd of split frequencies fell below 0.01 and the potential scale reduction factor statistics were approaching 1.0 (Ronquist *et al.*, 2012). For each dataset, trees from the first 25 % of sampled generations were removed as burn in and a majority rule consensus tree was estimated based on pooled post-burn-in trees.

Best-fit substitution models were selected using the Bayesian information criterion implemented in jModelTest version 2 (Darriba *et al.*, 2012). Maximum-likelihood (ML) phylogenetic analyses (Guindon *et al.*, 2010) were carried out as previously described using 1000 non-parametric bootstrap replications to assess support (Tang *et al.*, 2012). In all instances, tree topologies from Bayesian and ML analyses were similar; for brevity only the Bayesian trees are shown.

Consistent with a previous report (Wang *et al.*, 2013), two major groups of species of the genus *Bradyrhizobium* were evident in phylogenetic trees of 16S rRNA gene sequences: one represented by *Bradyrhizobium japonicum* and the other by *Bradyrhizobium elkanii* (Fig. S2). All 16 novel strains had identical 16S rRNA gene sequences and were placed in the phylogenetic group represented by *B. japonicum*. The sequence similarities of novel strains varied between 98.44 (*Bradyrhizobium denitrificans*) and 99.79 % (*Bradyrhizobium liaoningense*) relative to type strains of the 15 species in the *B. japonicum* group (Table S3).

MLSA of at least five housekeeping genes is used as a reliable method to define phylogenetic relationships and to identify novel lineages within the genus *Bradyrhizobium* (Rivas *et al.*, 2009; Tang *et al.*, 2012). The Bayesian phylogenetic tree of six concatenated housekeeping gene sequences (length 3210 bp) for the 16 novel strains and reference taxa is shown in Fig. 1. Consistent with the results of Tang *et al.*, (2012), the novel strains comprised three multilocus sequence types (STs) (represented by strains OO99^T^, OO85 and OM9) that were placed with high confidence (>95 % posterior probability) in a lineage that was distinct from named species of the genus *Bradyrhizobium*. Relative to OO99^T^, strains OO85 and OM9 had sequence similarities for the six concatenated housekeeping genes of >99.60 % whereas those for 19 named species varied between 88.35 and 95.42 % (Tables S3).

**Fig. 1.**
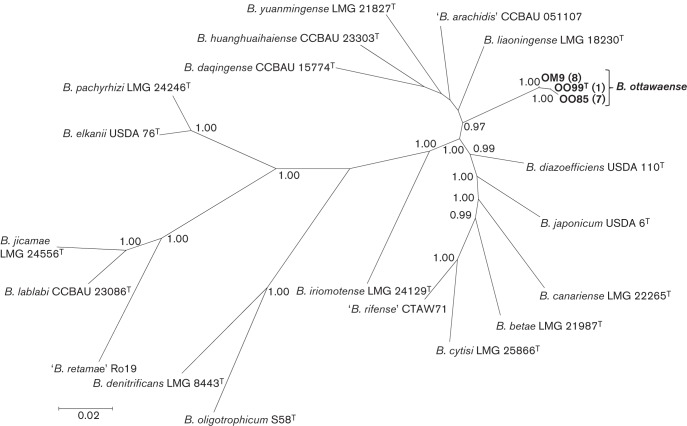
Bayesian phylogenetic tree of *atpD–glnII–recA–gyrB–rpoB–dnaK* concatenated gene sequences (3210 bp) for Bradyrhizobium *ottawaense* sp. nov. and reference taxa of the genus *Bradyrhizobium* (GTR+G+I substitution model). Only posterior probabilities ≥95 % are shown. Values in parentheses represent the number of strains in each of the three STs of *B. ottawaense*. Bar, expected substitutions per site.

To further analyse genetic differences between novel strains, we generated random amplified polymorphic DNA (RAPD) fingerprints for representative strains OO99^T^, OM9 and OO85 and reference taxa using four random primers (P1, P2, P3 and P5) and amplification methods described by Sikora *et al.* (2002). An example of the fingerprint profiles generated by one of the primers (P1) is shown in Fig. S3. A dendrogram based on the combined character matrix of fingerprint profiles generated by the four primers was reconstructed using upgma and the Dice coefficient implemented in GelCompare II software version 5.10 (Applied Maths). The three representative strains were readily distinguished and were placed in a single cluster separate from reference taxa (Fig. S4), consistent with their assignment to distinct STs, based on MLSA of protein encoding genes.

DNA–DNA hybridization experiments were performed as described by Willems *et al.*, (2001) using three representative strains (OO99^T^, OM9 and OO85) and relatives, *Bradyrhizobium yuanmingense* LMG 21827^T^ and *B. liaoningense* LMG 18230^T^. DNA–DNA hybridization values (Table S4) among the three novel strains varied between 73 % and 88 % whereas values for these strains hybridized with *B. liaoningense* LMG 18230^T^ and *B. yuanmingense* LMG 21827^T^ were between 23 % and 38 %. These data are consistent with the value of 70 % DNA–DNA relatedness that is considered the threshold for novel species definition Wayne *et al.*, (1987). The G+C mol% content of DNA for the three strains, determined by HPLC (Mesbah *et al.*, 1989), varied between 62.3 and 63.5 % which is within the range of DNA G+C content for the genus *Bradyrhizobium*.

Analysis of accessory genes encoding symbiotic functions (*nod* and *nif*) can provide useful information on the host range and specificity of symbiotic bacteria (Laguerre *et al.*, 2001). The phylogenetic tree of partial *nodC* sequences of novel strains and reference taxa is shown in Fig. S5. All 16 novel strains had identical *nodC* sequences and were placed in a group with five named species of the genus *Bradyrhizobium* that are symbiotic with soybeans. Sequence similarities of novel strains relative to these five species were ≥99.86 % (Table S3). Analyses of *nifH* gene sequences (Fig. S6 and Table S3) provided almost identical results.

Plant tests were carried out using Leonard jars as described by Tang *et al.*, (2012). The results (Table S5) show that representative strain, OO99^T^, elicited effective nitrogen fixing nodules (fix+) on *Glycine max*, *Glycine soja* and *Macroptilium atropurpureum*, partially effective nodules (fix±) on *Desmodium canadense* and *Vigna unguiculata*, and ineffective nodules (fix-) on *Amphicarpaea bracteata* and *Phaseolus vulgaris*.

For analysis of fatty acids, strains were grown on YEM agar at 28 °C. Bacteria were harvested and fatty acids extracted as described by Sasser (1990). Fatty acid identification was done using the Sherlock Microbial Identification System (MIDI) version 6.0 and the RTSBA6 database. Fatty acid profiles of three novel strains and reference taxa are shown in Table S6. The fatty acid profile of OO99^T^ was characteristic of the genus *Bradyrhizobium* with fatty acids 16 : 0 (12.83 %) and summed feature 8 (76.99 %) predominating (Tighe *et al.*, 2000). Five fatty acids (16 : 0, 16 : 1ω5*c*, 18 : 0 and summed feature 8) were common to all strains tested whereas seven (12 : 0, 14 : 0, 17 : 1ω8*c*, 18 : 1ω5*c*, 19 : 0 cyclo ω8*c* and summed feature 3) were detected in only some strains.

Phenotypic characteristics were further investigated using a variety of tests. Utilization of carbon and nitrogen sources was tested using YNB and YCB (Becton Dickinson) basal liquid media as described by Chahboune *et al.*, (2011) except that bromothymol blue was omitted. Cultures were incubated at 28 °C for 20 days on a rotary shaker when bacterial growth (turbidity) was recorded visually and using a Spectronic 21 UV spectrophotometer (Milton Roy) at 660 nm.

Tests of intrinsic antibiotic resistance were done according to Bromfield *et al.* (2010) using YEM agar medium amended with filter-sterilized solutions of the following antibiotics (Sigma Aldrich) at final concentration (µg ml^−1^): erythromycin (0, 50, 100, 200), penicillin G sodium salt (0, 10, 25), kanamycin sulphate (0, 25, 50, 100), tetracycline (0, 5, 10, 20), chloramphenicol (0, 25, 50), cefuroxime sodium salt (0, 5, 15, 30). Bacterial growth was recorded after 7 days at 28 °C. Tests for bacterial growth on YEM agar at 10, 28 and 37 °C, at pH 5 and pH 10 and in the presence of 1 % (w/v) NaCl were done as described by Xu *et al.*, (1995). The combination of phenotypic characteristics listed in Table 1 could be used to differentiate novel strains from recognized species of the genus *Bradyrhizobium*.

Based on data for genotypic and phenotypic analyses presented in this study, we propose that the novel group of 16 strains represent a novel species, named Bradyrhizobium *ottawaense* sp. nov.

## Description of Bradyrhizobium *ottawaense* sp. nov.

Bradyrhizobium *ottawaense* (ot.ta.wa.en′se. N.L. neut. adj. *ottawaense* of or belonging to Ottawa, Canada).

Cells are motile with subpolar flagella, Gram-stain-negative, aerobic, non-spore-forming rods (approx. 1.9 µm long and 0.8 µm wide). Colonies are circular, convex, translucent, beige, <1 mm diameter after 7 days at 28 °C on YEM agar medium. Mean generation time approx. 12–13 h in YEM broth at 28 °C. Produce an alkaline reaction on YEM agar. Growth occurs at pH 5–10 (optimum, pH 7.0). Growth is optimal at 28 °C but no growth occurs at 10 or 37 °C or in the presence of 1 % (w/v) NaCl. Utilizes d-mannose, d-xylose, l-arabinose, d-glucose, d-mannitol, maltose, l-rhamnose, l-proline, l-glutamic acid and l-threonine but not lactose, trehalose, succinic acid, l-sorbose, aspartic acid, glycine or dl-α-alanine. Resistance (µg ml^−1^) to antibiotics for representative strain OO99^T^: kanamycin (100), tetracycline (20), chloramphenicol (100), erythromycin (100), cefuroxime (<5), penicillin (<10). Summed feature 8 (18 : 1ω6*c* and/or 18 :1ω7*c*) and 16 : 0 are predominant fatty acids. Strain OO99^T^ elicits effective nodules on *Glycine max*, *Glycine soja* and *Macroptilium atropurpureum*, partially effective nodules on *Desmodium canadense* and *Vigna unguiculata*, and ineffective nodules on *Amphicarpaea bracteata* and *Phaseolus vulgaris*.

The type strain, OO99^T^ ( = LMG 26739^T^ = HAMBI 3284^T^), was isolated from an effective nodule of *Glycine max* in Ottawa, Ontario, Canada. The DNA G+C content of the type strain is 62.6 mol%.

## References

[r1] Altekar G. Dwarkadas S. Huelsenbeck J. P. Ronquist F. **(**2004**).** Parallel Metropolis coupled Markov chain Monte Carlo for Bayesian phylogenetic inference. Bioinformatics 20, 407–415 10.1093/bioinformatics/btg427 14960467

[r2] Bromfield E. S. P. Tambong J. T. Cloutier S. Prévost D. Laguerre G. van Berkum P. Tran Thi T. V. Assabgui R. Barran L. R. **(**2010**).** *Ensifer*, *Phyllobacterium* and *Rhizobium* species occupy nodules of *Medicago sativa* (alfalfa) and *Melilotus alba* (sweet clover) grown at a Canadian site without a history of cultivation. Microbiology 156, 505–520 10.1099/mic.0.034058-0 19875436

[r3] Chahboune R. Carro L. Peix A. Barrijal S. Velázquez E. Bedmar E. J. **(**2011**).** *Bradyrhizobium* *cytisi* sp. nov., isolated from effective nodules of *Cytisus* *villosus* in Morocco. Int J Syst Evol Microbiol 61, 2922–2927 10.1099/ijs.0.027649-0 21257682

[r4] Cole J. R. Wang Q. Fish J. A. Chai B. McGarrell D. M. Sun Y. Brown C. T. Porras-Alfaro A. Kuske C. R. Tiedje J. M. **(**2014**).** Ribosomal Database Project: data and tools for high throughput rRNA analysis. Nucleic Acids Res 42 (Database issue), D633–D642 10.1093/nar/gkt1244 24288368 PMC3965039

[r5] Darriba D. Taboada G. L. Doallo R. Posada D. **(**2012**).** jModelTest 2: more models, new heuristics and parallel computing. Nat Methods 9, 772 10.1038/nmeth.2109 22847109 PMC4594756

[r6] Garrity G. M. Bell J. A. Lilburn T. **(**2005**).** Family VII. Bradyrhizobiaceae fam. nov. In Bergey’s Manual of Systematic Bacteriology, 2nd edn, vol. 2, pp. 438 Edited by Brenner D. J. Krieg N. R. Staley J. T. Garrity G. M. New York: Springer

[r7] Guindon S. Dufayard J. F. Lefort V. Anisimova M. Hordijk W. Gascuel O. **(**2010**).** New algorithms and methods to estimate maximum-likelihood phylogenies: assessing the performance of PhyML 3.0. Syst Biol 59, 307–321 10.1093/sysbio/syq010 20525638

[r8] Hayat M. A. Miller S. E. **(**1990**).** Negative Staining, pp. 1–253 New York: McGraw-Hill

[r9] Hussey M. A. Zayaitz A. **(**2012**).** Endospore stain protocol. Wasinghton, DC: American Society for Microbiology http://www.microbelibrary.org

[r10] Itakura M. Tabata K. Eda S. Mitsui H. Murakami K. Yasuda J. Minamisawa K. **(**2008**).** Generation of *Bradyrhizobium japonicum* mutants with increased N_2_O reductase activity by selection after introduction of a mutated *dnaQ* gene. Appl Environ Microbiol 74, 7258–7264 10.1128/AEM.01850-08 18849448 PMC2592905

[r11] Laguerre G. Nour S. M. Macheret V. Sanjuan J. Drouin P. Amarger N. **(**2001**).** Classification of rhizobia based on *nod*C and *nif*H gene analysis reveals a close phylogenetic relationship among *Phaseolus vulgaris* symbionts. Microbiology 147, 981–993 11283294 10.1099/00221287-147-4-981

[r12] Mesbah M. Premachandran U. Whitman W. B. **(**1989**).** Precise measurement of the G+C content of deoxyribonucleic acid by high-performance liquid chromatography. Int J Syst Bacteriol 39, 159–167 10.1099/00207713-39-2-159

[r13] Powers E. M. **(**1995**).** Efficacy of the Ryu nonstaining KOH technique for rapidly determining gram reactions of food-borne and waterborne bacteria and yeasts. Appl Environ Microbiol 61, 3756–3758 7487012 10.1128/aem.61.10.3756-3758.1995 PMC167675

[r14] Rivas R. Martens M. de Lajudie P. Willems A. **(**2009**).** Multilocus sequence analysis of the genus *Bradyrhizobium*. Syst Appl Microbiol 32, 101–110 10.1016/j.syapm.2008.12.005 19201125

[r15] Ronquist F. Teslenko M. van der Mark P. Ayres D. L. Darling A. Höhna S. Larget B. Liu L. Suchard M. A. Huelsenbeck J. P. **(**2012**).** MrBayes 3.2: efficient Bayesian phylogenetic inference and model choice across a large model space. Syst Biol 61, 539–542 10.1093/sysbio/sys029 22357727 PMC3329765

[r16] Sasser M. **(**1990**).** *Identification of bacteria by gas chromatography of cellular fatty acids*, MIDI Technical Note 101. Newark, DE: MIDI Inc

[r17] Shields P. Cathcart L. **(**2012**).** Motility test medium protocol. Wasinghton, DC: American Society for Microbiology http://www.microbelibrary.org

[r18] Sikora S. Said R. Bradić M. **(**2002**).** Genomic fingerprinting of *Bradyrhizobium japonicum* isolates by RAPD and rep-PCR. Microbiol Res 157, 213–219 10.1078/0944-5013-00153 12398292

[r19] Tang J. Bromfield E. S. P. Rodrigue N. Cloutier S. Tambong J. T. **(**2012**).** Microevolution of symbiotic *Bradyrhizobium* populations associated with soybeans in east North America. Ecol Evol 2, 2943–2961 10.1002/ece3.404 23301163 PMC3538991

[r20] Tighe S. W. de Lajudie P. Dipietro K. Lindström K. Nick G. Jarvis B. D. W. **(**2000**).** Analysis of cellular fatty acids and phenotypic relationships of *Agrobacterium*, *Bradyrhizobium*, *Mesorhizobium*, *Rhizobium* and *Sinorhizobium* species using the Sherlock Microbial Identification System. Int J Syst Evol Microbiol 50, 787–801 10.1099/00207713-50-2-787 10758890

[r21] van Berkum P. Fuhrmann J. J. **(**2000**).** Evolutionary relationships among the soybean bradyrhizobia reconstructed from 16S rRNA gene and internally transcribed spacer region sequence divergence. Int J Syst Evol Microbiol 50, 2165–2172 10.1099/00207713-50-6-2165 11155993

[r22] Vincent J. M. **(**1970**).** A Manual for the PracticalSstudy of Root-Nodule Bacteria. Oxford: Blackwell Scientific

[r23] Wang R. Chang Y. L. Zheng W. T. Zhang D. Zhang X. X. Sui X. H. Wang E. T. Hu J. Q. Zhang L. Y. Chen W. X. **(**2013**).** *Bradyrhizobium arachidis* sp. nov., isolated from effective nodules of *Arachis hypogaea* grown in China. Syst Appl Microbiol 36, 101–105 10.1016/j.syapm.2012.10.009 23295123

[r24] Wayne L. G. Brenner D. J. Colwell R. R. Grimont P. A. D. Kandler O. Krichevsky M. I. Moore L. H. Moore W. E. C. Murray R. G. E. **& other authors (**1987**).** International Committee on Systematic Bacteriology. Report of the ad hoc committee on reconciliation of approaches to bacterial systematics. Int J Syst Bacteriol 37, 463–464 10.1099/00207713-37-4-463

[r25] Willems A. Doignon-Bourcier F. Goris J. Coopman R. de Lajudie P. De Vos P. Gillis M. **(**2001**).** DNA-DNA hybridization study of *Bradyrhizobium* strains. Int J Syst Evol Microbiol 51, 1315–1322 11491327 10.1099/00207713-51-4-1315

[r26] Wittwer J. W. **(**2014**).** Exponential growth rate in Excel. http://www.vertex42.com/ExcelArticles/exponential-growth.html

[r27] Xu L. M. Ge C. Cui Z. Li J. Fan H. **(**1995**).** *Bradyrhizobium liaoningense* sp. nov., isolated from the root nodules of soybean. Int J Syst Evol Microbiol 45, 706–711 10.1099/00207713-45-4-706 7547289

